# Development of a System to Monitor Laryngeal Movement during Swallowing Using a Bend Sensor

**DOI:** 10.1371/journal.pone.0070850

**Published:** 2013-08-05

**Authors:** Qiang Li, Kazuhiro Hori, Yoshitomo Minagi, Takahiro Ono, Yong-jin Chen, Jyugo Kondo, Shigehiro Fujiwara, Kenichi Tamine, Hirokazu Hayashi, Makoto Inoue, Yoshinobu Maeda

**Affiliations:** 1 Department of General Dentistry & Emergency, School of Stomatology, The Fourth Military Medical University, Xi'an, China; 2 Department of Prosthodontics, Gerodontology and Oral Rehabilitation, Osaka University Graduate School of Dentistry, Osaka, Japan; 3 Division of Dysphagia Rehabilitation, Niigata University Graduate School of Medical and Dental Sciences, Niigata, Japan; Weill Cornell Medical College, United States of America

## Abstract

**Background:**

Swallowing dysfunction (also known as dysphagia), which results in a deterioration of nutritional intake, slows rehabilitation and causes aspiration pneumonia, is very common following neurological impairments. Although videofluorographic (VF) examination is widely used for detecting aspiration, an objective and non-invasive method for assessing swallowing function has yet to be established because of a lack of adequate devices and protocols. In this paper, a bend sensor whose resistance is altered by bending was introduced to monitor swallowing-related laryngeal movement.

**Methods:**

Six healthy male volunteers were recruited in the present study. Specific time points on the signal waveform produced by the bend sensor were defined to describe laryngeal movement by differential analysis. Additionally, the physiological significance of the obtained waveform was confirmed by analyzing the sequential correlations between the signal waveform from the bend sensor and hyoid bone kinetics simultaneously recorded by VF.

**Results:**

Seven time points were successfully defined on the signal waveform to reference laryngeal movement. Each time point was well correlated with certain VF events, with evidence of no significant time lags, and there were positive correlations between waveform time points and matched VF events. Furthermore, obvious similarities were noticed between the duration of each phase on the signal waveform and the duration of the matched hyoid bone activity.

**Conclusions:**

The present monitoring system using a bend sensor might be useful for observing the temporal aspects of laryngeal movement during swallowing, and it was well coordinated with hyoid bone movement.

## Introduction

Swallowing refers to the entire process of deglutition that makes the food pass from the oral cavity to the pharynx and into the esophagus until it finally enters the stomach. Any difficulty in moving food from the mouth to the stomach is called swallowing dysfunction or dysphagia. Dysphagia often occurs following neurological impairments [1], [2], respiratory disorders [3], [4], diseases of the stomatognathic system due to tooth loss [5], [6], head and neck trauma [7], or cancer [8] across the lifespan, with effects ranging from difficulty in swallowing ingested substances to aspiration of food into the airways, resulting in malnutrition or even deterioration in quality of life (QOL). Sometimes, except in patients with structural defects, dysphagic complications such as aspiration may even appear before the anatomically intact patient recognizes difficulty in swallowing clinically. Therefore, it is important to evaluate swallowing function before problems arise.

A crucial event in the oropharyngeal swallow is the hyolaryngeal upward and forward excursion that contributes to laryngeal elevation [9]. Physically, it could have some correlation with tongue movement [10], and plays an important role in the tilt and seal of the epiglottis [11] as well as opening of the upper esophageal sphincter (UES) [12]. The combination of these actions displaces the larynx away from the trajectory of an oncoming bolus and allows the bolus to pass into the esophagus [13]. Thus, the ingested material cannot be aspirated or penetrate into the airway. Hyolaryngeal displacement is accomplished through the contraction of submental muscles (mylohyoid, geniohyoid, and anterior digastric) and the thyrohyoid muscle [14].

In clinical practice, several pieces of equipment have been used to assess swallowing function [15]–[18]. However, the best way is currently videofluorography (VF), which is a radiological technique based on the ingestion of a liquid or solid barium bolus. It is considered the gold standard because of its capability and reliability for observing associated structural events, especially the hyoid bone with its high-density image [19]. Unfortunately, VF is costly and exposes the patient to radiation. In addition, it is inconvenient in medically unstable patients, and the ingestion of a barium bolus can be very dangerous in patients at high risk of aspiration [20]. These unavoidable drawbacks greatly limit its wide application for sequential evaluations during diagnosis or follow-up studies.

Therefore, all of the above issues have prompted researchers to develop novel devices to assess swallowing function noninvasively. A piezoelectric sensor is such a device that is placed between the thyroid and cricoid cartilages to detect the mechanical upward and downward movements of the larynx during swallowing, and it has been proven useful in the study of the physiology of deglutition, as well as in its objective clinical evaluation in patients with dysphagia [21], [22]. Later, studies based on a piezoelectric acceleration transducer above the thyroid cartilage and two strain gauge pressure transducers easily demonstrated certain aspects of laryngeal elevation and coordination with tongue motion, but only the earliest laryngeal acceleration spike, without any other information related to laryngeal kinematics, was recorded [23]. Similar shortcomings also existed in an experiment with a device consisting of two sets of three pressure sensors to detect laryngeal movement [24]; the onset of laryngeal movement was defined as the time when the output voltage of the first sensor started to decrease.

In order to better understand actual laryngeal movement, simultaneous recording of laryngeal movement and videography was performed [25], [26]. Recently, some other researchers even took advantage of VF and videoendoscopy (VE) to observe the temporal aspects of swallowing [27], [28]. They successfully recorded the thyroid cartilage trajectory over time and related pharyngeal swallowing events, such as epiglottis activity and laryngeal closure. Though there is no denying that the sustained efforts have resulted in progress in the sensor field and have provided a better understanding of swallowing physiology, the sample number of such studies has been small [25], [27], [28], some appliances are complicated [25], and the position for fixing the sensor, such as a piezoelectric sensor [26], varies compared with early reports [21], [22]. In addition, hyoid bone movement, one of the most important and reliable markers during oropharyngeal swallow in VF examination [16], [29], has yet to be elucidated in detail by the sensors developed so far. Synchronicity with hyoid bone movement should be confirmed in the development of a sensing system for laryngeal movement.

In the medical field, bend sensors have been used for motion analysis. A bend sensor is a thin flexible membrane that changes resistance when bent; increasing the bend angle is generally associated with increased measured resistance. Researchers have applied it to obtain an approximate reconstruction of surface geometry [30] and measure finger activities [31], [32] in humans, considering it an alternative for recording dynamic changes or evaluating deliberate human acts. Accordingly, the aim of the present study was to develop a new and non-invasive monitoring system for laryngeal movement during swallowing with a bend sensor. After fixing the sensor along the midline of the front of the neck at the level of the prominence of the thyroid cartilage when it reaches the highest position during swallowing, the sequential correlations between the signal waveform produced by the bend sensor and hyoid bone activities simultaneously recorded by VF were found to verify the physiological significance of the obtained data during oropharyngeal swallowing.

## Methods

### Ethics statement

Written, informed consent was obtained from each participant after explanation of the aim and methodology of the study. The study protocols were approved by the Ethics Committee of Niigata University Faculty of Dentistry (No. 24-R7-05-22).

### Bend sensor design

**Figure 1:**
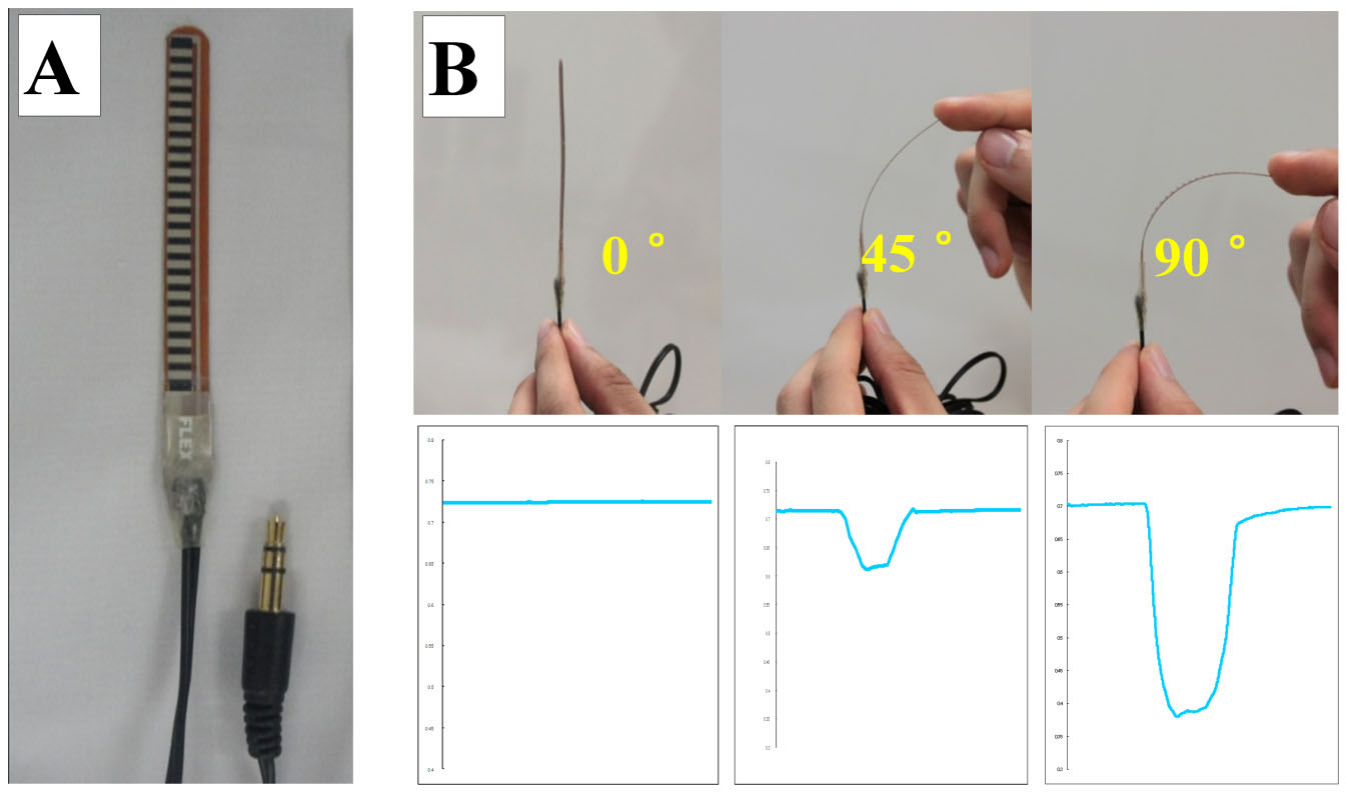
Overview of the bend sensor and produced waveforms. The resistance of the bend sensor changes when the metal pads (A) are on the outside of the bend. Deflection of the sensor may be achieved from 0° to 90°. The more it bends, the bigger the output voltage amplitude is (B).

The bend sensor (73.7 mm×6.4 mm×1.0 mm, MaP1783, Nihon Santeku Co. Ltd., Osaka, Japan) (Figure 1A) used in the present study works as a variable analogue voltage divider and flexes physically with motion. Its membrane construction is both resilient and somewhat durable. Inside the bend sensor are carbon resistive elements within a thin flexible substrate. When the metal pads (Figure 1A) are on the outside of the bend, it converts the change in bend to electrical resistance. There is a positive relationship between the changing resistance of the bend sensor and the amount of bend. The sensor then converts the resistance input to voltage using a dual-supply operational amplifier. As a result, the more the sensor bends, the greater is the output voltage amplitude (Figure 1B).

### Subjects and recording environment

Six adult male subjects (average ± S.D., 31.7±10.6 years) were recruited after providing their informed consent. None of the subjects reported a history of neurological diseases, musculoskeletal disorders, or masticating and swallowing problems. During measurement, the subject sat in an upright position, with the head supported by a headrest to avoid head retroflexion and to keep the Frankfort plane horizontal, with the feet touching the floor of the shielded VF room. Based on the result of preliminary experiment (see Appendix S1 and Figs. S1 and S2), the bend sensor was fixed on the skin surface along the midline of the neck in the position where the tip of the sensor was fixed to the skin at the level of the prominence of the thyroid cartilage when it reaches highest position during swallowing (see Fig. S1). The sampling frequency of the bend sensor was 1000 Hz, and the obtained signal was amplified (MaP1783PBA, Nihon Santeku Co. Ltd., Osaka, Japan) and stored on a personal computer through an interface board (PCI-3133A, Nihon Santeku Co. Ltd., Osaka, Japan).

A total of 5 mL of liquid barium (37°C) was inserted into the mouth by a syringe and kept on the mouth floor until the subject was instructed to swallow on cue in a single swallow. VF images were acquired in the sagittal plane using VF equipment (ULTIMAX80, Toshiba Co. Ltd., Tokyo, Japan). VF images at a speed of 30 frames per second were converted and recorded through the AD board (PowerLab ML880, AD Instrument); thus, the reliability limit of the timing measurement was 1/30th of a second (0.033 s). The trial was repeated six times for each subject, and a total of 36 swallows for the 6 participants was analyzed. The total exposure to radiation was limited to a maximum of 2 min per subject.

### Analysis of VF events during swallowing

Six VF events describing the movement of the hyoid bone are shown in Table 1. Consequently, five durations of hyoid bone movement were defined based on the VF events: #1, V1–V2: preparation phase of the hyoid bone while the hyoid bone moves slightly; #2, V2–V4: elevation phase of the hyoid bone while the hyoid bone moves upward from V2 to V3 and forward from V3 to V4; #3, V4–V5: stationary phase of the hyoid bone while the hyoid bone maintains the most superior-anterior position; #4, V5–V6: descending phase of the hyoid bone while the hyoid bone moves to the original position; and #5, V2–V6: active phase of the hyoid bone.

**Table 1:** Definitions of VF events observed by VF.

VF events	Physiological activity of the hyoid bone
V1	Onset of slight movement of the hyoid bone
V2	Onset of upward movement of the hyoid bone
V3	Onset of forward movement of the hyoid bone
V4	Onset of stationary phase of the hyoid bone
V5	Offset of stationary phase of the hyoid bone
V6	Offset of movement of the hyoid bone

V1 to V6 indicate VF event 1 to VF event 6.

### Differential analysis of the laryngeal movement signal from the bend sensor

From a series of previous studies, laryngeal movement during swallowing was recognized to be well coordinated and similar with hyoid bone movement; it starts from a rapid upward displacement that is then followed by anterior displacement, stays at the most superior-anterior position, and finally restores to the original position [15], [25], [26], [29]. Many trials to read such laryngeal movement in the signal waveform were done using different kinds of monitoring systems, and the expression of laryngeal movement on the signal waveform depended on the sensing procedure. As for the bend sensor, the location and direction of the bending force changed with laryngeal movement during swallowing; hence, the signal waveform showed complex sequential changes, which made it difficult to evaluate the state of laryngeal movement based solely on the original signal waveform. Therefore, several time points on the signal waveform were defined as references for describing the state of laryngeal movement based on the change in magnitude and direction of velocity and smoothed acceleration signals that could be obtained by first-order differentiation and averaging 50 sequential second-order differentiated values of the original signal, respectively.

### Analysis of sequential correlations between laryngeal and hyoid bone movements

The sequential correlation between laryngeal and hyoid bone movements was analyzed by comparing the timing of each VF event with that of each time point defined on the signal waveform from the bend sensor on the common time series. Independent *t*-tests were performed to compare the differences in the time courses of each time point of the waveform and the matched VF event, as well as the time durations from the bend sensor and VF, while the intra-class correlation coefficient was used to evaluate the correlations of waveform time points and VF events. All data are expressed as the means ± standard deviation (S.D.). A P-value less than 0.05 was considered significant. Statistical analyses were performed with SPSS 16.0 software (SPSS Inc., Chicago, IL, USA).

## Results

### Time points on the waveform defined by the differential analysis of signals from a bend sensor

**Figure 2:**
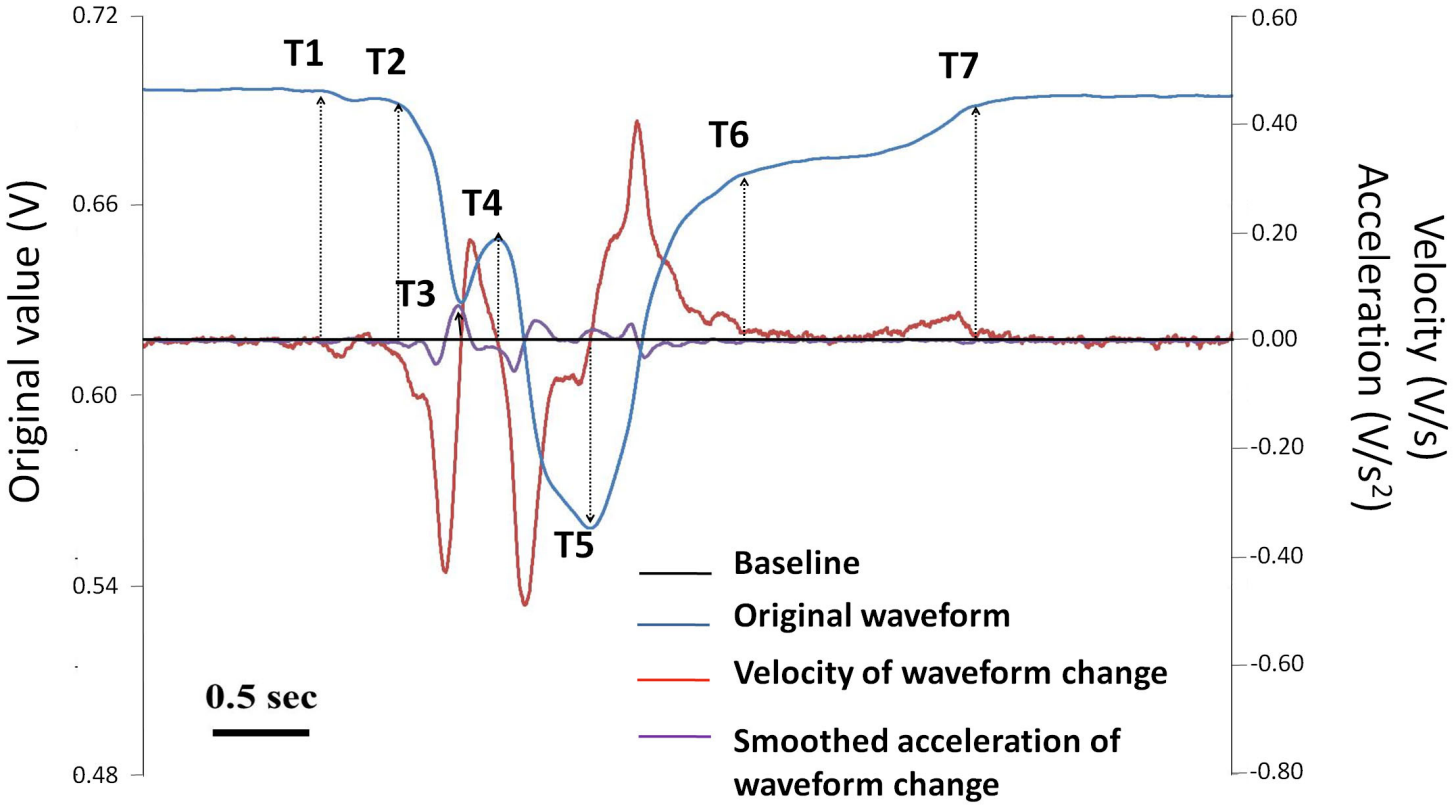
Time points on the signal waveform from the bend sensor defined by first- and second-order differentiation of the signal. The black line indicates the baseline, the blue line indicates the original signal waveform, the red line indicates the first-order differentiated value (the velocity of waveform change), and the purple line indicates the averaged 50 sequential second-order differentiated values (the smoothed acceleration of waveform change). Bar = 0.5 second.

The sensor produced a “V”-shaped waveform, with a preliminary movement at the beginning, followed by rapid downward movement with a small and obvious notch until reaching its peak point. Then, the waveform reversed, quickly at first and then slowly after a turning point. Finally, it returned to the baseline (Figure 2). For analyzing the signal from the bend sensor, seven time points were defined on the original waveform based on the first-order differentiated signal (velocity of waveform change) and the averaged 50 sequential second-order differentiated values (smoothed acceleration of waveform change) (Figure 2, Table 2). Accordingly, five waveform durations were used thereafter: #1, T1–T2: preliminary phase of the waveform while bending of the sensor started at T1 and increased slightly until the onset of rapid movement (T2); #2, T2–T4: the first part of the downward phase of the waveform while bending of the sensor increased rapidly from T2 to the onset of the notch (T3), then decreased until the offset of the notch (T4); #3, T4–T5: the second part of the downward phase of the waveform while bending of the sensor increased rapidly again from T4 to the peak of the waveform (T5); #4, T5–T6: recovery phase of the waveform while bending of the sensor decreased from T5 to the turning point within the recovery phase (T6); and #5, T2–T6: the major region of the waveform.

**Table 2:** Definitions of time points of the signal waveform produced by the bend sensor in position A.

Time points	Time points of the waveform	Definition with differential method
T1	Onset of slight movement of the waveform	Defined by the velocity when it deviates from the baseline
T2	Onset of rapid movement of the waveform	Defined by the onset of smoothed acceleration in the expected area
T3	Onset of the notch	Defined by the cross point of velocity and baseline
T4	Offset of the notch	Defined by the cross point of velocity and baseline
T5	Peak time of the waveform	Defined by the cross point of velocity and baseline
T6	Turning point time during recovery phase of the waveform	Defined by the offset of smoothed acceleration in expected area
T7	Offset of the waveform	Defined by the velocity when it reaches baseline

### Sequential correlations between each time point on the signal waveform from the bend sensor and VF events

**Figure 3:**
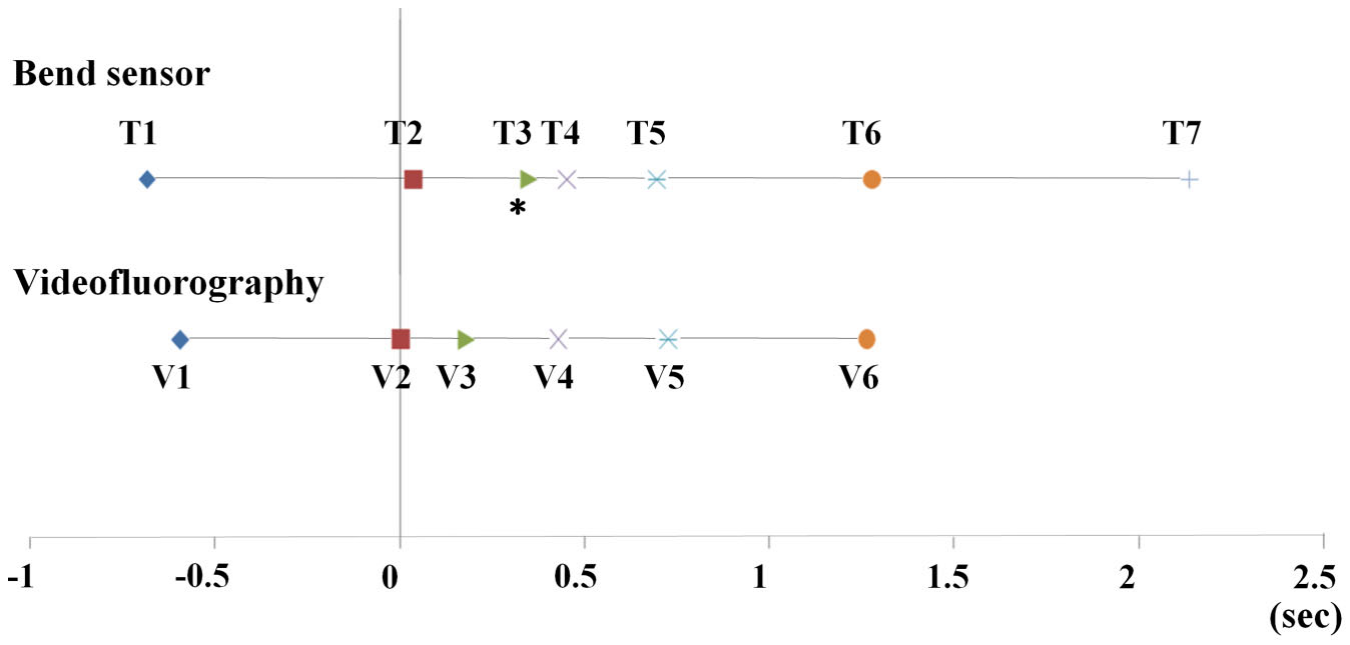
Time points (T1–T7) on the signal waveform from the bend sensor and VF events (V1–V6) on the common time series. T1 to T6 indicate time points 1 to 6. V1 to V6 indicate VF event 1 to VF event 6. The timing of V2 was set to 0 s. * *p*<0.05 vs. V3. Since T7 was recorded last as the offset of the waveform, it was not compared with any VF events.

Sequential correlations between time points on the signal waveform from the bend sensor (T1–T7) and VF events (V1–V6) were investigated on the common time series with the timing of V2 set at 0 s (Figure 3, Table 3). There were no obvious time lags between T1 and V1 (*P* = 0.452), T2 and V2 (*P* = 0.381), T4 and V4 (*P* = 0.760), T5 and V5 (*P* = 0.669), or T6 and V6 (*P* = 0.948). However, T3 appeared significantly later than V3 (*P* = 0.020). Since T7 is the offset of the waveform that was recorded last, it was not compared with any VF events.

**Table 3:** Comparisons of time points (T1–T7) on the signal waveform from the bend sensor and VF events (V1–V6) on the common time series.

Time points	Time (s)	VF events	Time (s)	p
T1	−0.682±0.303	V1	−0.594±0.318	0.452
T2	0.034±0.091	V2	0.000±0.000	0.381
T3	0.350±0.129	V3	0.181±0.076	0.020
T4	0.451±0.139	V4	0.430±0.087	0.760
T5	0.695±0.101	V5	0.725±0.134	0.669
T6	1.277±0.307	V6	1.266±0.190	0.948
T7	2.137±0.377			

T1 to T6 indicate time points 1 to 6. V1 to V6 indicate VF event 1 to VF event 6. The timing of V2 was set to 0 s. Since T7 was recorded last as the offset of the waveform, it was not compared with any VF events.

**Figure 4:**
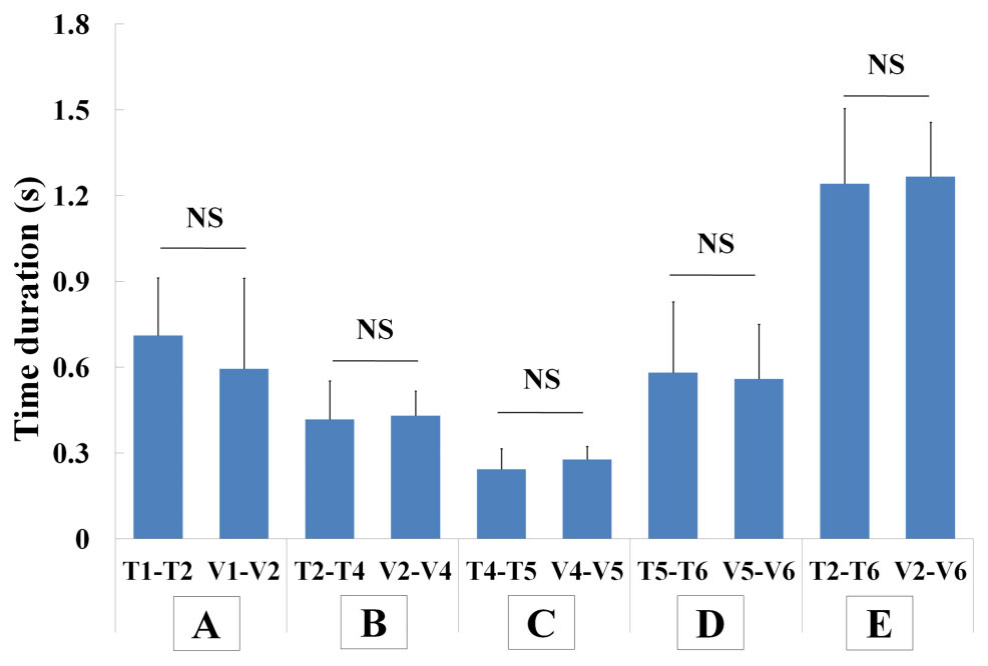
Comparisons of duration of time points and VF events. A indicates the comparison of T1–T2 and V1–V2, B indicates the comparison of T2–T4 and V2–V4, C indicates the comparison of T4–T5 and V4–V5, D indicates the comparison of T5–T6 and V5–V6, and E indicates the comparison of T2–T6 and V2–V6. NS, *p*>0.05.

Positive correlations were observed between waveform time points and VF events with the intra-class correlation coefficients after setting V2 to 0 s. Of note, significant correlations were found between T1 and V1 (r = 0.663, *p*<0.001), T4 and V4 (r = 0.849, *p*<0.001), T5 and V5 (r = 0.609, *p*<0.001), and T6 and V6 (r = 0.381, *p* = 0.010) (Table 4). Results of comparisons between each phase on the signal waveform from the bend sensor and the VF events are shown in Figure 4. The preliminary phase of the waveform was longer but with no significant difference in comparison with the preparation phase of the hyoid bone (T1–T2: 0.711±0.201 s, V1–V2: 0.594±0.318 s, *p* = 0.384; Figure 4A). The first part of the downward phase of the waveform was similar to the elevation phase of the hyoid bone (T2–T4: 0.417±0.135 s, V2–V4: 0.430±0.087 s, *p* = 0.848; Figure 4B). The second part of the downward phase of the waveform was also similar to the stationary phase of the hyoid bone (T4–T5: 0.243±0.072 s, V4–V5: 0.278±0.045 s, *p* = 0.350; Figure 4C). Likewise, similar results were seen between the recovery phase of the waveform and the descending phase of the hyoid bone (T5–T6: 0.582±0.247 s, V5–V6: 0.558±0.192 s, p = 0.859; Figure 4D). In addition, obvious similarity was found between the major region of the waveform and the active phase of the hyoid bone (T2–T6: 1.242±0.263 s, V2–V6: 1.266±0.190 s, *p* = 0.859; Figure 4E).

**Table 4:** Correlations between time points of the signal waveform and VF events with V2 set to 0 s.

	*r*	*p*
T1 and V1	0.663	<0.001
T4 and V4	0.849	<0.001
T5 and V5	0.609	<0.001
T6 and V6	0.381	0.01

T1 to T6 indicate time points 1 to 6. V1 to V6 indicate VF event 1 to VF event 6.

**Figure 5:**
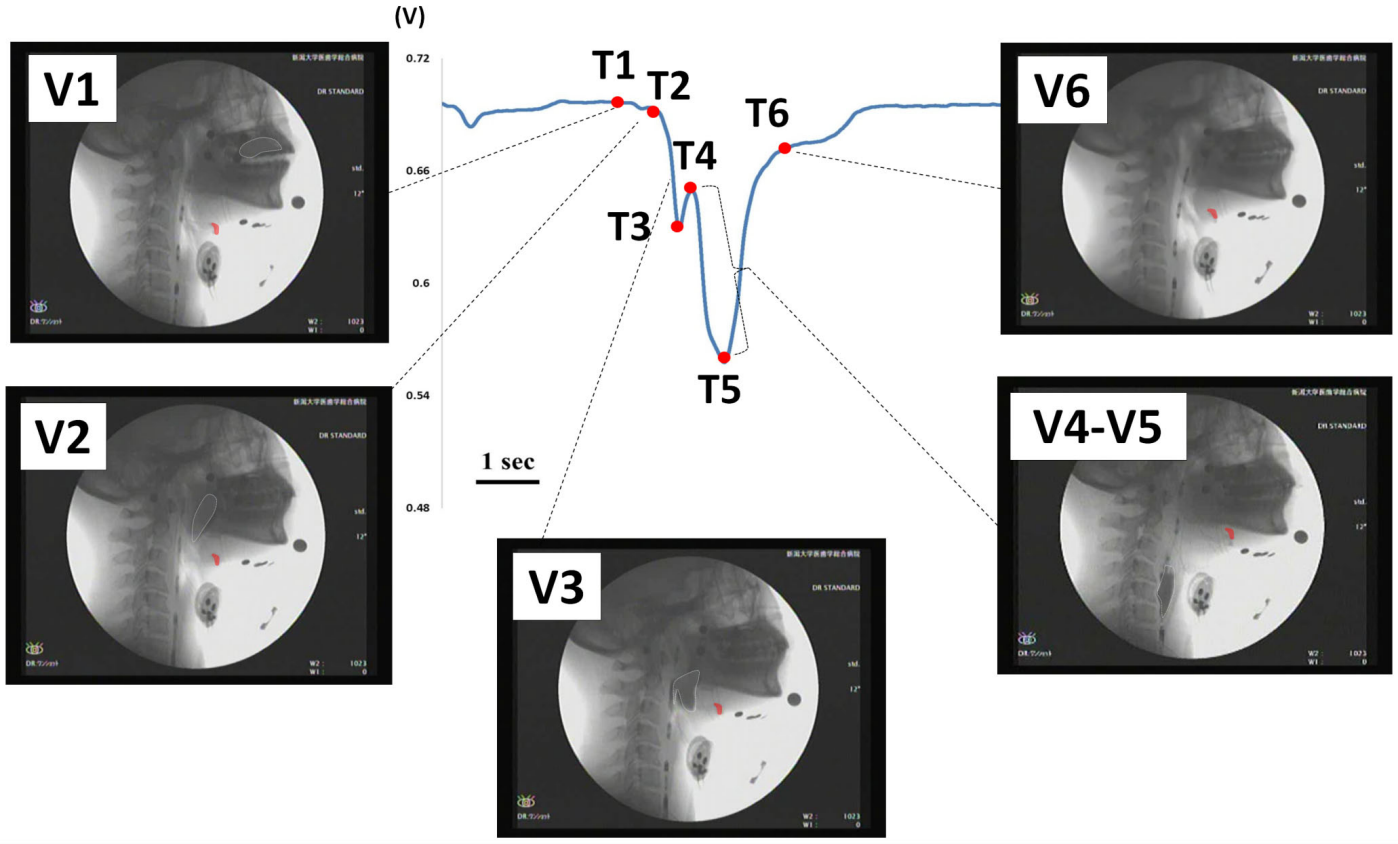
Sequential correlations between time points on the signal waveform from a bend sensor (T1–T6) and VF events (V1–V6). T1 to T6 indicate time points 1 to 6. V1 to V6 indicate VF event 1 to VF event 6. The silhouette of the hyoid bone is marked in red. The bolus is marked with a white dotted line. Bar = 1 second.

Sequential correlations between time points on the signal waveform and VF events suggested by the above data analysis are illustrated in Figure 5. The starts of slight movement (V1) and rapid upward movement of the hyoid bone (V2) were synchronized with those of slow (T1) and fast increases of bending (T2), respectively. Then, the start of forward movement of the hyoid bone (V3) preceded the onset of the notch (T3), and the duration while the hyoid bone kept the most superior-anterior position (V4–V5) was nearly coincident with the duration from the offset of the notch (T4) to the peak of the waveform (T5). Finally, the timing when the hyoid bone returned to the original position (V6) was synchronized with the end of a rapid decrease in bending (T6).

## Discussion

In the present study, a novel system to monitor laryngeal movement during swallowing with a bend sensor was developed. After determining the most suitable position for the bend sensor on the frontal neck (see Appendix S1), seven time points on the signal waveform the bend sensor were defined to describe laryngeal movement by differential analysis, and then, the correlations and co-ordinations between time points on the signal waveform and VF events were confirmed. These step-by-step procedures demonstrated that this system is a practical monitoring tool of hyolaryngeal excursion during swallowing and thus has the potential to facilitate the early diagnosis of dysphagic patients.

In the present study, only young males were selected for measurement. First, structural features such as the sizes of the thyroid and cricoids cartilages are very different between males and females, which may affect the results. Second, some studies reported differences in some parameters of swallowing behaviors between men and women [33]–[37]. Because the aim of this study was to establish the validity of identifying laryngeal movement with bend sensor, we selected only young healthy men to avoid a possible sex bias in the results.

The possibility of inter-rater error was undeniable in the previously developed monitoring systems for laryngeal movement, because laryngeal movement was directly read by the characteristic change on the signal waveform recognized by the rater. Therefore, in the present study, laryngeal movement was evaluated on the signal waveform based on the time points defined by the changes in the magnitude and direction of velocity and smoothed acceleration of sensor bending (Figure 2). To the best of our knowledge, this is the first monitoring system in which reading of the signal waveform of laryngeal movement was objectified by mathematical analysis. This guarantees that the correlations between time points and VF events found in the next step are not affected by the raters' subjective judgments.

A series of analyses on the correlations between the above time points and VF events on the common time series with V2 set to 0 s (Figures 3 and 4, Table 3) clearly revealed sequential coordination between the produced waveform and hyoid bone activities, which is summarized in Figure 5. Overall, it is consistent with the report by Parmer that strong positive correlations exist between displacements of the larynx and hyoid bone [15]. The present results showing similar time durations measured with the bend sensor and VF simultaneously indicated that some phases of hyoid bone movement during swallowing, not only the stationary phase (V4–V5), but also the preparation phase (V1–V2), the elevation phase (V2–V4), the descending phase (V5–V6), and the total active phase (V2–V6) could be evaluated by the bend sensor (Figure 4). These findings give us the insights necessary to estimate some hyoid bone activities during swallowing using the data obtained from the bend sensor.

There were very small time lags between some time points from the bend sensor and matched VF events (Figure 3). In the findings, slight movement of the hyoid bone (V1), which has also been demonstrated by Hiiemae et al. [38], was close to the onset of slight movement of the signal waveform (T1), indicating the high sensitivity of the bend sensor. Physically, the thyroid cartilage activities are because of thyrohyoid muscle contraction [12], [39], [40], while the suprahyoid muscle contributes to hyoid bone movement [14], [19]. Electromyogram recordings have revealed that the activity of the thyrohyoid muscle appears a little later than the onset of the suprahyoid muscle activity [40], which may be the explanation for the small but not significant delay of the onset of rapid downward movement of the signal waveform (T2) and the offset of the notch (T4) compared with the onset of rapid movement of the hyoid bone (V2) and the onset of the stationary phase of the hyoid bone (V4), respectively (time lags between T2 and V2 and between T4 and V4 were 0.034 s and 0.021 s, respectively). However, it is difficult to explain the physiological background of the current result that the onset of forward movement of the hyoid bone (V3) occurred in the first part of the downward phase of the signal waveform (T2–T4) and obviously earlier than the onset of the notch (T3). Changes in the direction of bending velocity within the notch (from T3 to T4) on the signal waveform might be caused by the change in the thyroid cartilage movement following that in hyoid bone movement, but it could not be confirmed in the experimental setting.

It was also found that the time lags between the peak time of the signal waveform (T5) and the offset of the stationary phase of the hyoid bone (V5), as well as the turning point of the signal waveform (T6) and the offset of movement of the hyoid bone (V6), were small enough to help us assume some important hyoid bone activities (0.030 s and 0.011 s, respectively). With regard to the results, the downward motion of the signal waveform from T4 to T5 suggests that the thyroid cartilage is still moving during the stationary phase of the hyoid bone. This interesting phenomenon might be explained by the finding of Mepani et al. [41] that the maximum thyrohyoid muscle shortening occurred close to the time of maximal superior-anterior hyoid excursion. Obviously, as the thyrohyoid muscle does not contract during the early phase but shows great activity during the later phase of superior laryngeal movement, the spatial relationship between the hyoid bone and thyroid cartilage must be stable.

However, the data were obtained from healthy male participants in the current study, and the sample size was small. Additionally, the motions of the tongue and some deeper structures, such as the soft palate, pharyngeal walls, and UES, could not be analyzed via the waveform produced by the bend sensor. Therefore, we plan to use the bend sensor in more subjects, including female subjects and individuals with dysphagia, to further confirm its general applicability. The dynamic coordination between the tongue and hyolarynx will also be evaluated by carrying out tongue pressure measurements and bend sensor measurements concurrently. We hope that the bend sensor will play an auxiliary role to VF to draw the entire picture needed to evaluate swallowing function in the near future.

## Conclusions

A new system for monitoring laryngeal movement using a bend sensor was developed. This simple and maneuverable system has promise clinically in the objective, non-invasive, and quantitative evaluation of the timing and displacement of hyolaryngeal excursion during oropharyngeal swallowing. It has the potential to be an auxiliary method to VF in the diagnosis of dysphagia in anatomically intact patients. However, the system configuration should be improved for studies of different applications in the future.

## Supporting Information

Appendix S1 **Summary of the preliminary experiments for deciding the position for fixing a bend sensor on the frontal neck skin.**
(DOCX)

Figure S1 **Three positions of bend sensor on the frontal neck.** In position A, the tip of the sensor was fixed to the skin at the level of the prominence of the thyroid cartilage when it reaches highest position during swallowing; in position B, the tip of the sensor was fixed to the skin at the level of the prominence of the thyroid cartilage at rest; in position C, the tip of the sensor was fixed to the skin at the level of the coniotomy region between the thyroid cartilage and cricoid cartilage.
(TIF)

Figure S2 **Typical signal waveforms from a bend sensor attached at three positions in one subject.** Figures A, B, and C indicate the waveforms produced in positions A, B, and C, respectively. Figure D indicates the frequency of similar wave patterns in different positions. Figure E indicates the amplitude of the produced waveforms in different positions. Bar = 1 second. * *p*<0.05, ** *p*<0.01. N = 12.
(TIF)
