# Supplementary material for: Development of a System to Monitor Laryngeal Movement during Swallowing Using a Bend Sensor
Source: PLoS One. 2013 Aug 5;8(8):e70850. doi: 10.1371/journal.pone.0070850 (PMC3733966; doi:10.1371/journal.pone.0070850)
Supplement: Appendix S1 — Summary of the preliminary experiments for deciding the position for fixing a bend sensor on the frontal neck skin. (DOCX) [file pone.0070850.s001.docx]

**Appendix S1**

Three positions of the bend sensor on the front of the neck were tested in 12 healthy adult male subjects (average ± S.D., 27.2 ± 1.6 years) to identify the best position to obtain a clear and stable signal of laryngeal movement during swallowing. The bend sensor was fixed on the skin surface along the midline of the neck in three positions (positions A, B, and C; Fig. S1) by palpation. Then, 5 mL of water (37ºC) was inserted into the mouth by a syringe and kept on the mouth floor until the subject was instructed to swallow on cue in a single swallow. The position was measured randomly (www.researchrandomizer.org), and five repetitions were performed by each subject in a position on the same day.

The ratio of the number of subjects with similar waves to the total number of subjects (N=12) was defined as the frequency of similar wave patterns and used to indicate reproducibility. The maximum amplitude of the waveform was calculated via the absolute value of subtracting the baseline from the peak value. Those two parameters were compared among the three positions to identify the best position.

Generally, the bend sensor produced a simple “V”-shaped waveform in position A (Fig. S2A), an irregular “V”-shaped waveform in position B (Fig. S2B), and a reversed “V”-like-shaped waveform in position C (Fig. S2C). The waveform showed good intra-individual repeatability in all positions. The frequency of similar wave patterns among subjects differed little, i.e., 91.67% (11 of 12 subjects) in position A, and 75% (9 of 12 subjects) in the other two positions, with no significant difference (p=0.264, [chi-square](http://dict.youdao.com/w/chi-square/) [test](http://dict.youdao.com/w/test/)s) (Fig. S2D). However, the maximum amplitude of the waveform in position A (0.101 ± 0.045 V) was much larger than that in position B (0.044 ± 0.018 V) or C (0.034 ± 0.011 V) (p=0.012 and p=0.004, respectively; repeated one-way ANOVA with the Bonferroni correction, Fig. S2E). In addition, the flat baseline of the signal waveform produced in position A enabled us to observe slight movement of the waveform, while vibrating movements that existed in positions B and C were puzzling (Figs. S2A, B, and C). As these results indicated that the waveform produced in position A was simple, reproducible, and clear, position A was recognized as suitable for fixing the bend sensor and was used in the following experiments.
