# Supplementary figures and images for: Development of a System to Monitor Laryngeal Movement during Swallowing Using a Bend Sensor
Source: PLoS One. 2013 Aug 5;8(8):e70850. doi: 10.1371/journal.pone.0070850 (PMC3733966; doi:10.1371/journal.pone.0070850)

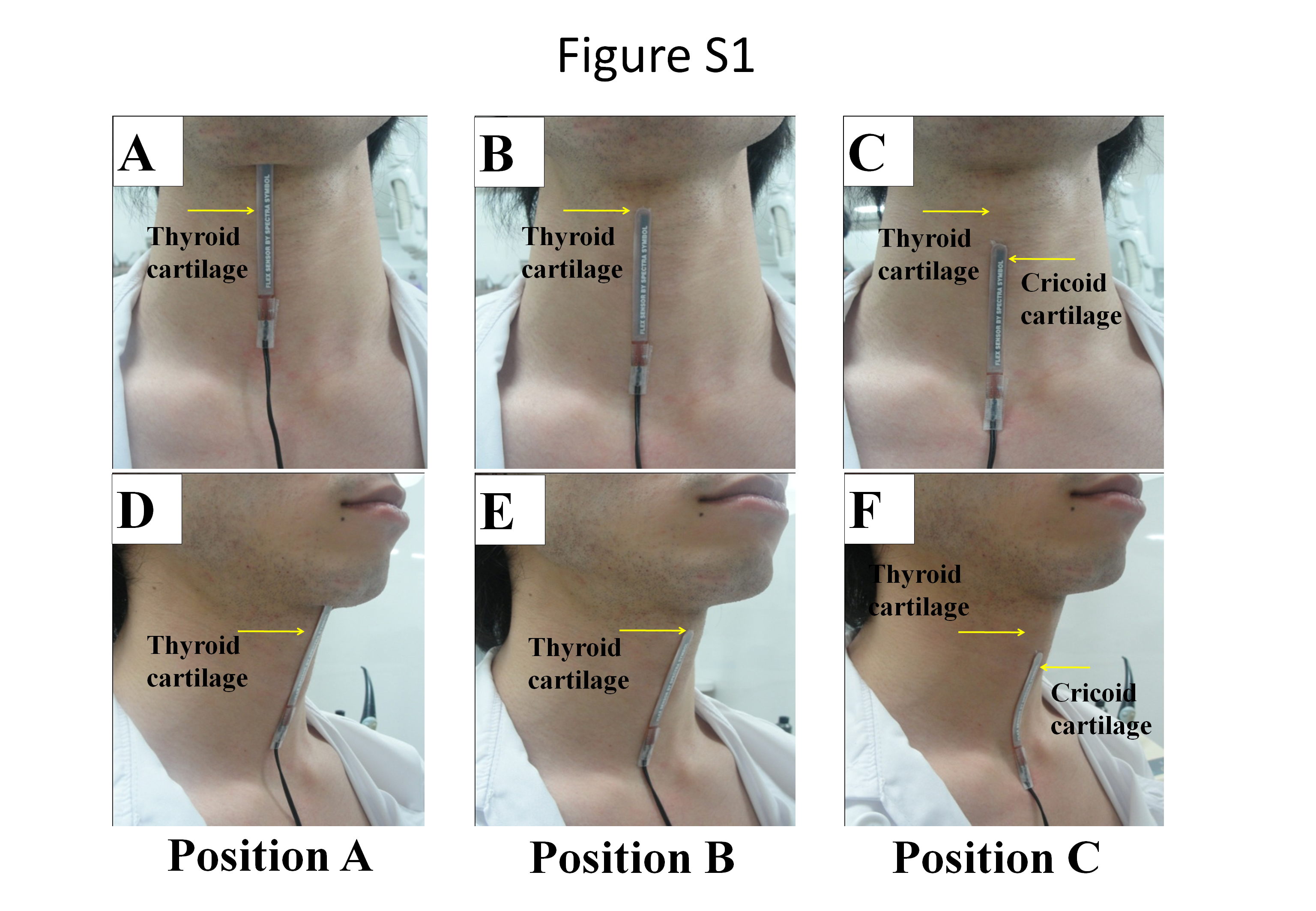

Supplement: Figure S1 — Three positions of bend sensor on the frontal neck. In position A, the tip of the sensor was fixed to the skin at the level of the prominence of the thyroid cartilage when it reaches highest position during swallowing; in position B, the tip of the sensor was fixed to the skin at the level of the prominence of the thyroid cartilage at rest; in position C, the tip of the sensor was fixed to the skin at the level of the coniotomy region between the thyroid cartilage and cricoid cartilage. (TIF) [file pone.0070850.s002.tif]

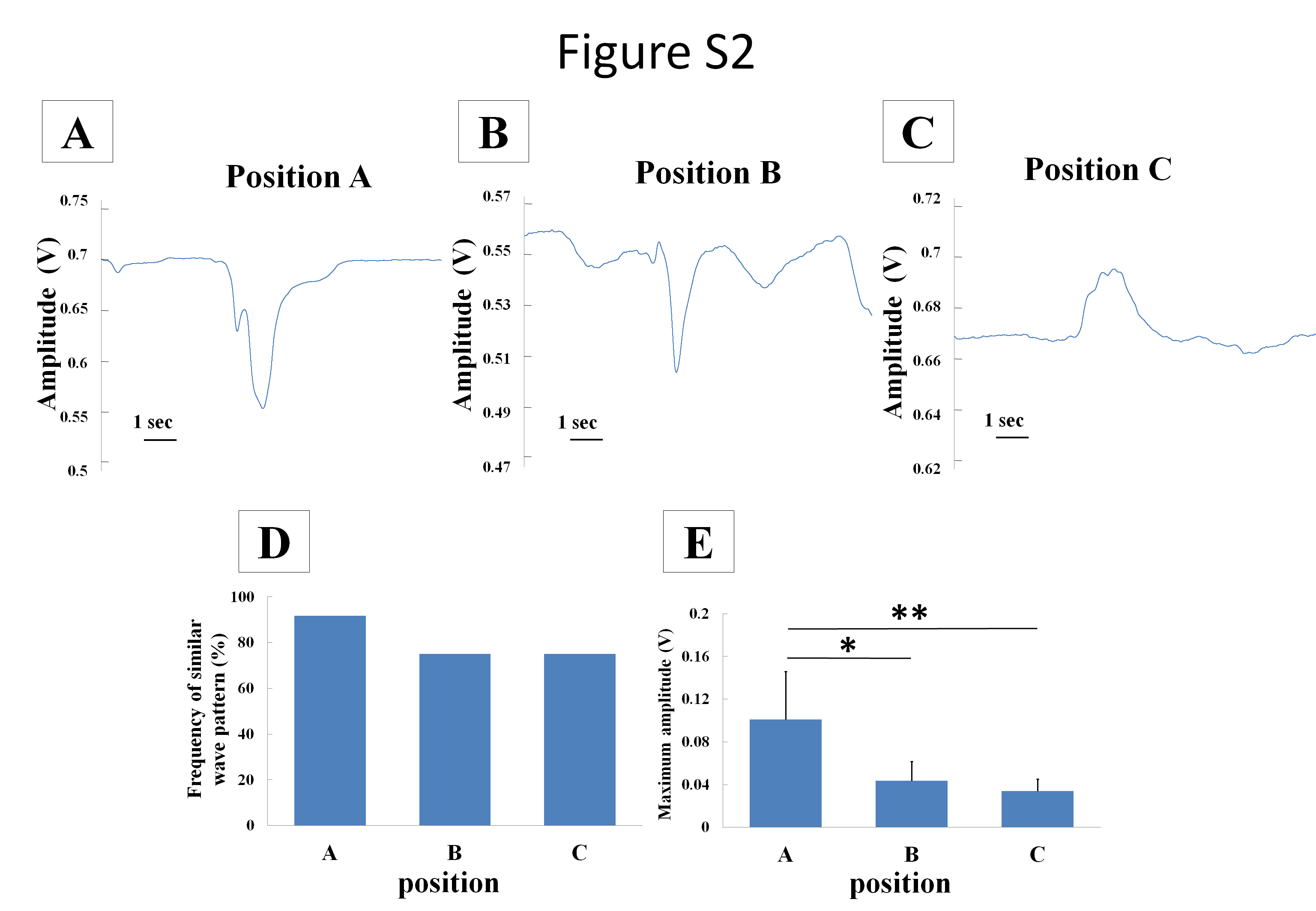

Supplement: Figure S2 — Typical signal waveforms from a bend sensor attached at three positions in one subject. Figures A, B, and C indicate the waveforms produced in positions A, B, and C, respectively. Figure D indicates the frequency of similar wave patterns in different positions. Figure E indicates the amplitude of the produced waveforms in different positions. Bar = 1 second. * p<0.05, ** p<0.01. N = 12. (TIF) [file pone.0070850.s003.tif]
